# Does descriptive text change how people look at art? A novel analysis of eye - movements using data -driven Units of Interest

**DOI:** 10.16910/jemr.10.4.4

**Published:** 2017-11-22

**Authors:** Alan Davies, Manuele Reani, Markel Vigo, Simon Harper, Clare Gannaway, Martin Grimes, Caroline Jay

**Affiliations:** School of Computer Science, University of Manchester, United Kingdom; Manchester Art Gallery, United Kingdom

**Keywords:** Art, paintings, eye tracking, eye movement, painting narration, art perception, areas of interest, regions of interest, Markov chain

## Abstract

Does reading a description of an artwork affect how a person subsequently views it? In a controlled study, we show that in most cases, textual description does not influence how people subsequently view paintings, contrary to participants' self-report that they believed it did. To examine whether the description affected transition behaviour, we devised a novel analysis method that systematically determines Units of Interest (UOIs), and calculates transitions between these, to quantify the effect of an external factor (a descriptive text) on the viewing pattern of a naturalistic stimulus (a painting). UOIs are defined using a grid-based system, where the cell-size is determined by a clustering algorithm (DBSCAN). The Hellinger distance is computed for the distance between two Markov chains using a permutation test, constructed from the transition matrices (visual shifts between UOIs) of the two groups for each painting. Results show that the description does not affect the way in which people transition between UOIs for all but one of the paintings -- an abstract work -- suggesting that description may play more of a role in determining transition behaviour when a lack of semantic cues means it is unclear how the painting should be interpreted. The contribution is twofold: to the domain of art/curation, we provide evidence that descriptive texts do not effect how people view paintings, with the possible exception of some abstract paintings; to the domain of eye-movement research, we provide a method with the potential to answer questions across multiple research areas, where the goal is to determine whether a particular factor or condition consistently affects viewing behaviour of naturalistic stimuli.

## Introduction

Science and art are often considered to be parallel disciplines with little interaction between the two ( 1 ); here we provide a scientific perspective on the perception of art, which emerged from a collaborative project between the University of Manchester and Manchester Art Gallery. Manchester Gallery were specifically interested in understanding the behaviour of their web visitors for curatorial purposes. We explored whether reading a description of an artwork affects the way a person subsequently views it in a controlled study, leading to a richer understanding of how people view art, and a generalizable method that can be used by researchers in eyemovement research. The method presented can help to answer similar questions about differences in viewing behaviour between groups, when using stimuli where Areas of Interest (AOI) segmentation is challenging.

**Figure 1.**
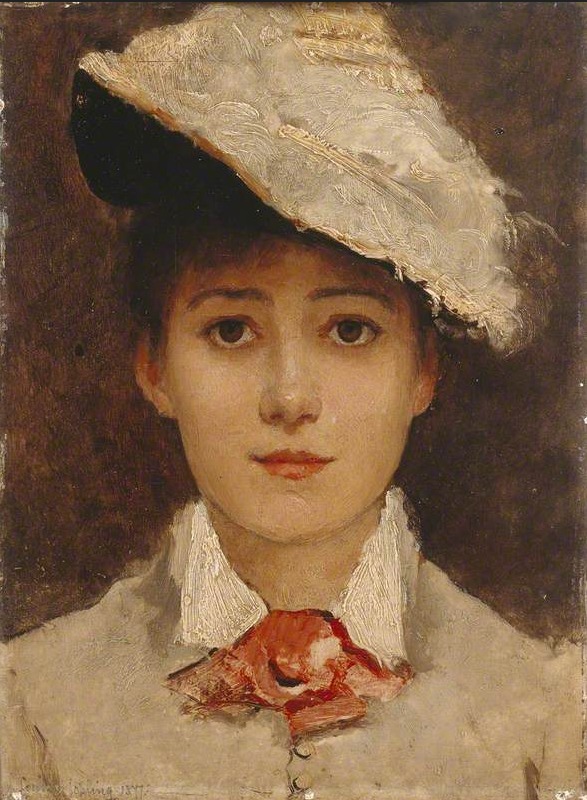
‘Self-Portrait’, by Louise Jopling (1843-1933).

Art is a unique and subjective perceptual experience( 1 ). Although arguably the best context for some forms of art, museums can be difficult for some people to visit, including older people, those who have disabilities, and those who are unable to travel to them. It is also known that the time people spend viewing artworks decreases as people move through an exhibition, a phenomenon termed “museum fatigue” ( 2 ). As it is now possible to view many paintings online, more people can potentially access artworks than previously, and can access those works faster. It is known that the context in which art is viewed has an effect on how people evaluate it ( 3 ). With more and more art consumed online, new questions are emerging as to how best to digitally present it. Eyetracking can play a valuable role in understanding how people perceive art, and has the potential to provide information that can be used to support its curation. Quantitatively analysing gaze data over artwork can be challenging, due to the fact that images are often naturalistic (representing the various colours and forms as they appear in nature), and do not generally contain explicit semantic regions that can be labelled as Areas/Regions of Interest (AOIs/ROIs). In this paper we introduce a method of stimulus segmentation for subsequent data analysis that reduces researcher bias and aids in the segmentation of stimuli with difficult to identify or subjective semantic details. The method is used to quantitatively examine whether presenting a descriptive text to people before they view a painting subsequently affects their viewing pattern. The text consists of a short description written by a curator or other expert, providing information about the painting; in the current study, we used texts taken from the Art UK website ( http://www.artuk.org ), which accompany each painting displayed online. The following example is taken from one of the paintings used in the study, entitled, ‘Self Portrait’, by Louise Jopling: Figure 1

“A frontal bust portrait of the artist as a young woman with her hair tied up, wearing a pale coat with white collar and matching hat, set at an angle. At her neck she wears a decorative pink neck scarf. Her skin and features are smoothly and evenly painted, in comparison to her more textured clothes. She is set against a dark plain background.”

This study is the first to consider the impact of a descriptive text on subsequent gaze patterns over a painting. As visual scanning is the genesis of aesthetic experience ( 4 ), we apply a quantitative method to determine if the presence or absence of a description has any impact on the visual behaviour of participants by using eye-tracking, an established measure of visual attention ( 5 ) that has previously been identified as a meaningful method for quantifying how people view artworks ( 1 ).

The texts examined in the current study are primarily used for describing the stimulus such that it can be searched for within an online collection. We demonstrate that reading such descriptions does not generally appear to affect people's viewing behaviour in terms of the nature of fixation frequency or duration, and that whilst transition behaviour between UOIs is generally similar across groups, it appears to vary more when a work is abstract.

## Background

Areas of interest (AOIs) are used to identify semantic regions in a stimulus that are of importance to an experiment ( 6 ). It can be challenging to apply these to artwork, due to the non-uniform and naturalistic nature of the stimuli, which make it harder to determine how and where to draw the boundaries for AOIs. Our work addresses this issue by segmenting the image into regions using a data-driven clustering algorithm, before going on to compare differences in gaze transitions between these areas across two groups. We begin with a review of other work that has used eye-tracking to explore how people view art, and highlight the effect of the environment on how a work is perceived. The second section looks at different methods of segregating images to produce areas of interest, to provide context for our approach.

### Art and eye-tracking

Several studies have used eye-tracking to investigate how people view and interact with art. Bubic, Susac and Palmovic ( 7 ) used eye-tracking to explore how people view images that can represent either a human face, or alternately when inverted (displayed upside down), a still-life image, where no distinct facial components are identifiable. The results showed that in the upright position people fixated more on the image elements that represent faces, focusing more on the eyes (upper AOI) than in the inverted position.

Gartus, Klemer, and Leder ( 8 ) considered the effect that context has on how people view art, using eye tracking to determine whether perception changed according to whether works were viewed in a museum or street context. They demonstrated that viewing durations were substantially longer in the museum context than they were in the street context. The study also provided evidence that the context had no impact on ratings of graffiti art, but that modern art received higher ratings for beauty and interest in the museum context.

The effect of context on the “experience” of viewing art has also been considered by Brieber et al. ( 2 ), who determined that viewing art in the context of a museum, as opposed to a laboratory setting, led people to view paintings for longer, and that there was a stronger relationship between viewing time and appreciation in the laboratory context. In both situations the information labels were viewed longer for works with a higher appreciation rating.

In a study that explored centre-stage effect (CSE) -the phenomenon that items/options placed in the central position are more popular than those located to the sides - participants were shown three paintings in a row, and asked to select their preferred painting. Findings show that allocation of a substantially larger proportion of gaze to the paintings in the left and centre positions was not associated with preference when the paintings were identical. The fixation duration did, however, predict preference when the paintings were different. The centre-stage effect was seen in the centre image only when the paintings were identical and had a positive valence. They conclude that valence has a greater impact on CSE than gaze allocation.

The authors suggest that the ‘centre stage heuristic’ - the assumption that the best items are in the centre - can explain their results. The final fixation was found to be predictive of people choosing the central item, if it exhibited positive valence ( 9 ). As acknowledged by the authors, only a subset (22 of 50) participants had their eyemovements measured with an eye-tracker, which may have had an impact on the relationship strength between gaze allocation and preferred painting ( 9 ).

Massaro et al. ( 4 ) found that visual exploration patterns appeared to be affected more by knowledge-driven top-down processes when people are viewing faces, than when they are viewing natural scenes, where the gaze path appears to be driven more by low-level features.

Visual behaviour can also be manipulated by modulating the luminance of a painting, to guide people's gaze to a portion of the painting not being directly focused upon ( 10 ).

Aesthetic experience is known to be comprised of competing top-down and bottom-up processes ( 4 ). A large variability between participants (n=10) viewing figurative paintings was identified by Quiroga and Pedreira ( 1 ), in a study examining how digital manipulation of artworks affects fixation patterns. This variability, attributed to the participants’ individual knowledge and appreciation of the work, made analysis of the subject difficult ( 1 ) .

Image complexity has also been considered in relation to gaze-behaviour. Complexity relating to pattern detail that changes with scale can be examined with the fractal dimension. Regions of paintings with a higher fractal dimension were fixated on for a longer period than other regions with a lower fractal dimension ( 11 ).

Many studies do not use AOIs when analysing gaze over artwork, choosing instead to employ qualitative methods (e.g. observation of gaze plots or heat maps) or statistical methods, for understanding basic distribution of gaze ( 2, 3, 9 ).

A study by Brinkmann et al., ( 12 ) looked at differences in the attention profiles of participants when looking at both abstract and representational artwork. The study revealed more diffuse attention for abstract art. They also found that eye-movement patterns varied more for the abstract paintings than the representational paintings, pointing to individual image characteristics playing a greater part in structuring attention when compared to socio-demographic factors. The study used a bottom-up approach to define AOIs, which were defined by circles with an area of 90 pixels and a minimum of 5 fixations in the circle per minute.

One study ( 13 ) did examine the effect that a painting title had on non-realistic cubist paintings. In this study there were 3 experimental conditions: 1) no title; 2) participants had to decide on a title; 3) participants told the actual title. They discovered that the duration of fixations increased in the group told the painting title relative to the group tasked with coming up with a title for the painting. They also found that the most fixated area for all the paintings was the centre of the painting. There was an increase in saccadic amplitude for the group that were told the title of the paintings in the case of one painting, which was attributed to additional cognitive processing being required to link the title to the image. The study concluded that the title information did have an impact on the eye-movements and fixation distribution over time ( 13 ). For this study the AOIs were defined for each painting by arbitrarily splitting the image into a grid containing 12 cells, the rationale for which is not discussed in detail in the paper.

Here we consider the impact of painting description, written by experts, on the gaze behaviour of people viewing artwork.

### Defining areas of interest

The generation of areas or regions of interest requires researchers to make decisions about how to segment a stimulus. As AOIs usually correspond to semantic items in a scene, they can be very useful for determining which of these items participants focus their interest upon ( 6 ). AOIs defined by the researcher in a top-down manner can be very useful for answering particular research questions, but they are also subject to bias, as a decision about how to segment the scene will affect the subsequent data analysis process, and may not be optimal. Although it is not possible to eradicate all of the top-down factors that can affect the way people view scenes, such as the semantic dependency of objects or the context of the scene ( 5 ), it is possible to reduce the potential bias introduced by researcher-imposed segmentation of the scene for analysis purposes. Gridded AOIs are crude in comparison, but allow for content-independent analysis to take place ( 14 ). One of the principal issues with using gridded AOIs is determining the cell size, as this can significantly affect the results (i.e. capture more or fewer fixations in the defined geospatial area).

Bottom-up AOIs have been generated with clustering techniques, using circles and a minimum number of fixations to define the AOI ( 12 )( 12, 15 ). This is also the case for eye-tracking analysis software, such as Eyetrace ( 16 ) that allows both user defined top-down AOIs and bottomup data-driven AOIs using fixation clustering. This is achieved by setting neighbourhood thresholds or using mean-shift clustering. As circles do not tessellate there are gaps between them that exclude fixation data. Where the circles do overlap and do not leave spaces, deciding which cluster fixations belong to which AOIs can be problematic. This can also be compounded by differently sized AOIs that make carrying out comparative analysis challenging. Indeed Klein, ( 15 ) state that they did not analyse the AOI data across paintings due to differences in their gross geometric structure.

Other methods used to segment stimuli include Voronoi diagrams, fuzzy AOIs and convex hulls. Voronoi diagrams (segmentation of an area into different regions, derived from the distance between predefined points in subsets of the area) divide scenes into cells. The distribution of the cells correlates to the fixation density distributions. This method is predominantly spatial and is analogous to fixation clustering ( 17 ).

Fuzzy AOIs do not have hard borders, and thus rather than taking a ‘hit or miss’ approach, they use a probabilistic method to determine which AOI (or none) a fixation belongs to ( 6 ). Fuzzy AOIs can also be of use when data quality is lower, as thresholds can be varied to account for poorer precision ( 6 ). Convex hulls, which are sets of points in Euclidean space or on a plane can be used to describe the minimum area covered by a cluster of fixations. The convex hull essentially represents the line that encapsulates a set of these points such that the enclosing polygon is convex as opposed to concave (i.e. has no indents). Holmqvist et al. ( 6 ) point out that generating AOIs this way is unsuitable for transitional analysis due to the amount of manual post editing that would be required and the potential for inflated values (when compared to smaller AOIs), caused by the collection of stray data points. The irregularly shaped and sized AOIs resulting from Voronoi diagrams and convex hulls makes quantitative comparison between them complex, and difficult from a statistical perspective.

Orquin, Ashby, and Clarke ( 18 ) describe several recommendations for using AOIs with behavioural eyetracking studies. These recommendations include using maximum margins around AOIs when there are large distances between objects of importance on the stimulus. This allows fixations related to the object to be included and reduces overlap. By contrast, if the distance between objects is small, a smaller AOI margin should be used. Orquin, Ashby, and Clarke ( 18 ) go on to state that researchers should either choose these AOI margins beforehand based on the possible overlap, or alternatively do this post-hoc based on data conforming with quality criteria. Finally, they suggest that details of the AOI margins are reported alongside the analysis.

Like ( 12 ) and ( 15 ), we use a bottom-up clustering approach, but rather than using this to generate the AOIs directly, we instead apply clustering to determine the cell size for a grid that we then apply to each painting. Details of our approach to AOI definition are provided in the ‘Analysis’ section below.

## Methods

A between-subjects experiment was conducted. The main factor was “stimulus presentation”. This had two levels: 1) “no-textual description”, henceforth referred to as the “no-description” condition, where 8 paintings were shown sequentially without any description, and 2) “description” condition, where the same 8 paintings were presented sequentially, preceded by a descriptive narrative written by experts presented before each painting. The order of the presentation of the paintings was fixed, and the same for both conditions. Participants were randomly allocated to one of the two conditions. Neither of the groups were told the titles of the paintings or given any information concerning the artists. No specific task(s) were given to the participants, allowing them to view the paintings naturally.

### Procedure

The experiment was run at two open day events at the University of Manchester, in a quiet controlled environment, in facilities dedicated to the purpose. Participants were given an information sheet to read, and asked to sit in front of a desktop computer with a Tobii X2-60 (https://www.tobiipro.com/siteassets/tobii-pro/usermanuals/tobii-pro-x2-60-eye-tracker-usermanual.pdf/?v=1.0.3 ) eye-tracker, attached to a monitor with a resolution of 1366 x 768 pixels.

Forty-four participants (with normal or corrected-to-normal vision) who attended the open days volunteered to take part in the experiment. Two participants were excluded due to poor data quality leaving 42 participants, 24 males, 18 females. Before starting the experiment participants signed an informed consent form describing the nature of the study, in accordance with the University's ethical procedures.

Once the participant's gaze had been calibrated, they began the experiment. In the ‘no-description’ condition, the paintings were displayed on the screen in sequence for 10 seconds each, and the participant sat and viewed them. In the ‘description’ condition, a written description of the painting appeared on the screen; when the participant had read this, he or she pressed the space bar to view the subsequent painting (also for 10 seconds per painting). Participants in the description condition were also asked a multiple choice on-screen question after viewing all the paintings: “Do you think the text (information about the paintings) changed the way you looked at the paintings?” (yes/no). Participants' gaze was recorded throughout the experiment.

### Stimuli

Digital versions of eight paintings were selected by Manchester Art Gallery staff as being representative of their collections, and artwork they would be interested in understanding people's visual perception of. The descriptive text for each painting was obtained from the “Art UK” website ( http://www.artuk.org/discover/artworks/search/venue:\\manchester-art-gallery-7282-54853 ). The paintings consisted of 3 landscapes, 2 portraits and 3 abstract pieces. The descriptions of the paintings were written by art experts table 1.

**Table 1.**
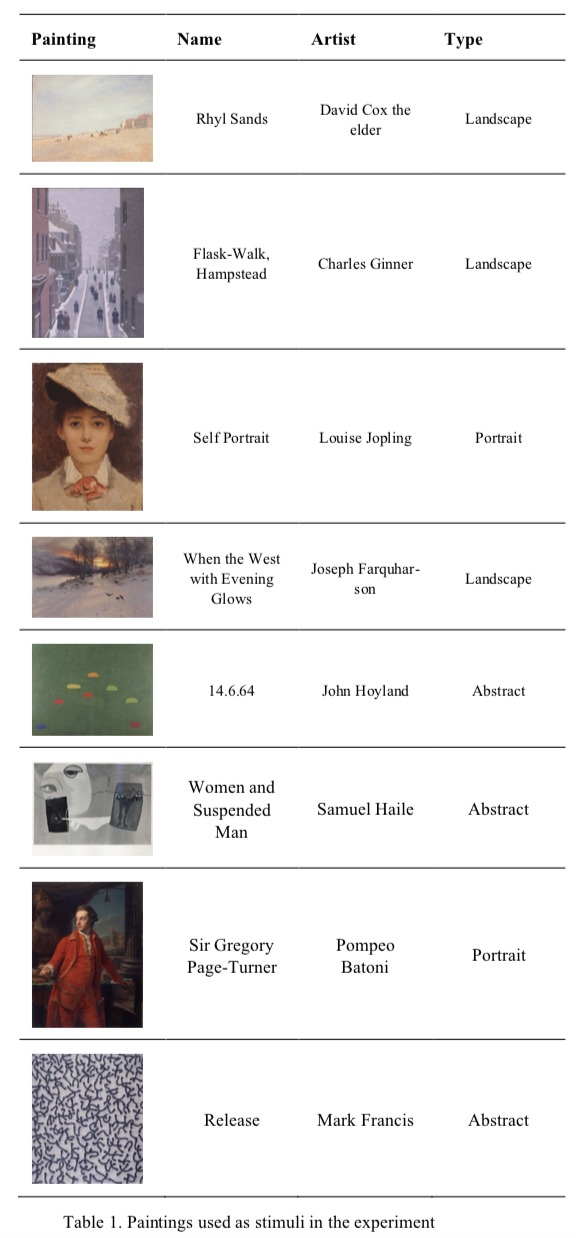
Paintings used as stimuli in the experiment

## Analysis

All the analysis reported here was carried out using the R project for statistical computing, version 3.3.2. ( 19 ). Note that where effect size (partial eta squared) is reported, it was calculated on untrimmed data. The full code and data is available from ( 20 ). The Densitybased spatial clustering of applications with noise (DBSCAN) algorithm ( 21 ) was used to cluster visual fixations for each of the paintings, using the DBSCAN package ( 22 ) available for R. The algorithm was selected as it is widely used for cluster discovery, without a requirement to state the number of clusters in advance. This was important, as we did not know *a priori* where the fixations would be clustered, or how many clusters there would be. Density is determined by counting the points in a specific radius (termed Eps). Where the number of these points exceeds the threshold defined by a value called MinPts, it is considered a “core point” by the algorithm. Noise points are those that are neither core points, nor contain a core point within the Eps radius ( 21 ). Formally the Eps-neighbourhood (Eps) for a point is defined as N_Eps_(P) = {q ∈ D | dist(p,q) ≤ Eps}. Points can also be directly density-reachable, defined as p ∈ N_Eps_(q) and | N_Eps_ (q)| ≤ MinPts ( 21 ). The optimal Eps value was selected for each painting by computing the k-nearest neighbour distances and plotting them in ascending order to visualise the “knee of the curve” (point of curve with significant change) that corresponds to the optimal Eps value. The MinPts value, referring to the minimum number of points that are required to form “core points”, was set to 4. This value was used as the original authors state that k distance graphs did not alter significantly with values > 4, but did, however, require greater computational effort ( 21 ).

A grid of squares was then applied to each painting, with the cell dimensions (height and width) set as double the average of the optimal DBSCAN Eps value (radius). Each of the cells represented a *Unit of Interest* (UOI), for which fixation data was calculated.

**Figure 3:**
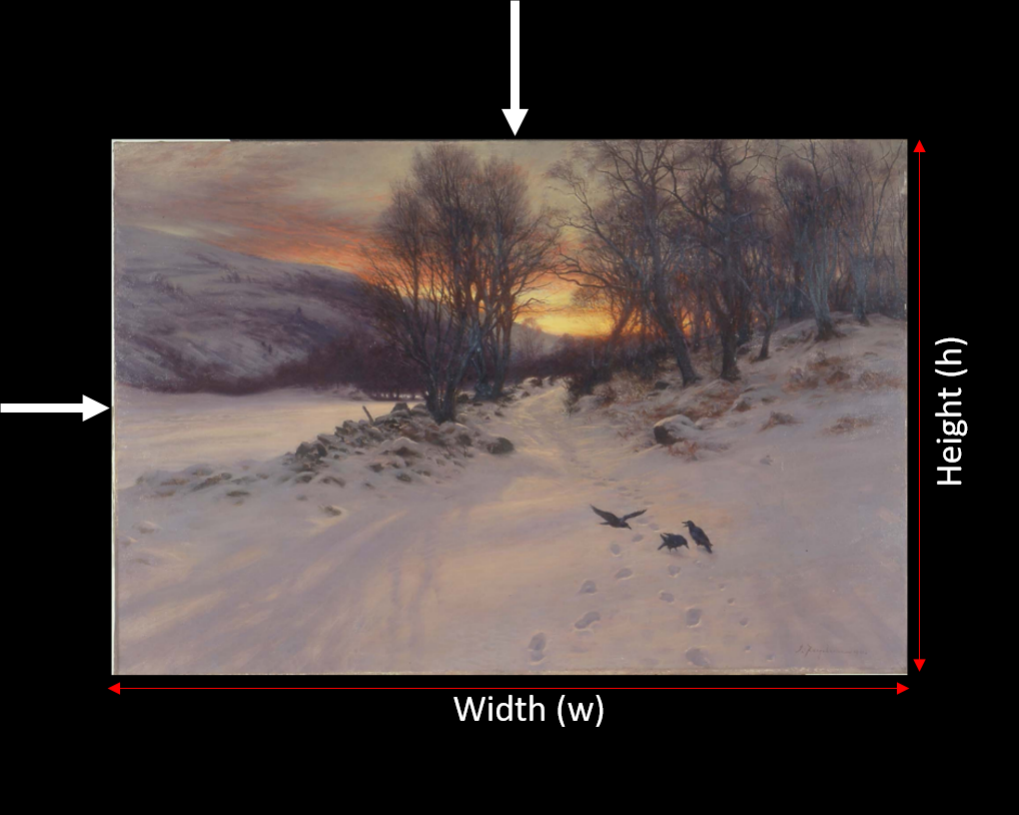
Horizontal and vertical offsets applied to locate the grid in the area occupied by the paintings

To ensure the analysis considered only fixations on the painting itself, we added an offset to the grid using a bespoke algorithm to calculate the position of the painting inside the black container area Figure 3. Additionally as the division of individual cell dimensions into the image space may leave a remainder, we accounted for this by locating the grid in the centre of the image so that any additional space between the area of the grid and that of the painting will be around the edges of the painting. This was done instead of locating the grid in the top left of the image on the assumption that the salient features for a given painting are located centrally rather than peripherally.

**Figure 4:**
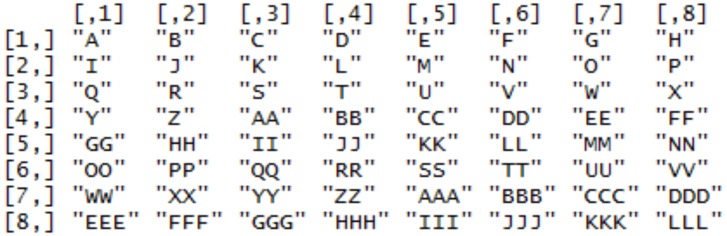
Sample UOI's generated for the “Self-portrait” painting. The 8x8 grid generates 64 UOIs for this painting

As there is an inherent error in gaze accuracy for each eye-tracker, we consider the appropriateness of the size of the cells used in the grid to determine if the cell size is small enough to be impacted significantly by gaze accuracy error. The units spanned by the visual field (x) can be calculated by first determining the visual angle (Φ) given the object size (s) and the object distance (d) converted from radians into degrees (Equation 1).

**Figure:**
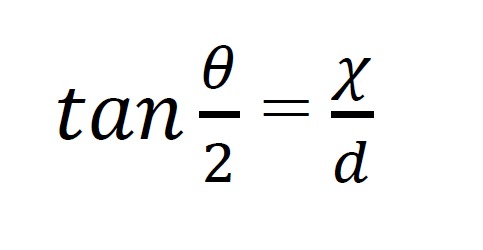


**Figure:**
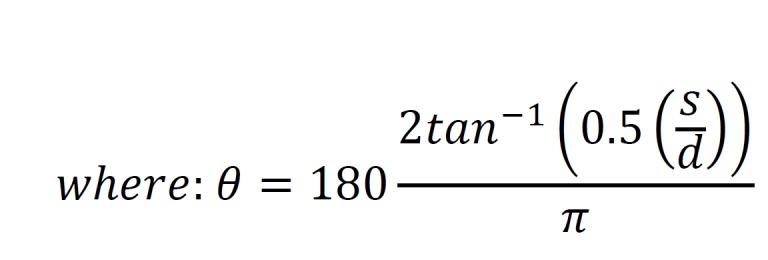


The smallest of the cell sizes used in this study (1.55°) is greater than the 1 to 1.5° that is suggested as the minimum practical AOI size ( 6 ). A transition matrix was then constructed for each condition (description and no-description), representing the number of transitions from and to each cell in the grid. This is then converted into a Markov chain representing the probability of transitioning from a given UOI to the same UOI, or to a different one. This technique has been used to compare differences between clinicians making correct and incorrect interpretations of medical scans with the Jensen-Shannon distance ( 23 ), and modified by Reani, ( 24 ) to use the Hellinger distance, which is more appropriate for comparing transition behaviour, as it permits values of 0 in the transition matrix. The Hellinger distance (Equation 2) is used to determine the difference between the Markov chains representing each condition and can be used as a proxy for dissimilarity.

**Figure:**
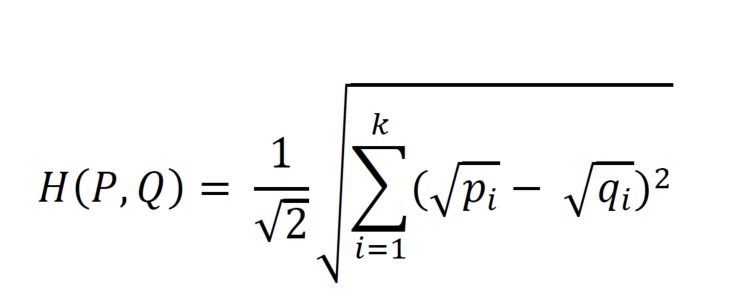


This value can then be compared against that obtained for two groups of equivalent size, but containing participants chosen at random. By performing this operation 10,000 times using a permutation test ( 25 ), we obtain a distribution of the difference between groups compared chosen at random, which against the difference can then between be the description and no-description groups. This allows a threshold to be set, where “p-values” to the right of the critical value allow the rejection of the null hypothesis. This allows us to see if there is something “special” about the two conditions that is unlikely to be explained by random chance, given that permutation tests are known to be robust against Type I error ( 26 ).

The individual participants' scanpaths were also compared against one another for both conditions (description and no-description). The order in which the participants transition their gaze around the UOIs was represented as a “string” of text. This essentially represents each participant's scanpath around the stimulus in terms of the sequences of UOIs visited. Although this does not account for the temporal dimension of the scanpath sequence, it does allow comparison between the areas visited. The Levenshtein distance applies a cost for each operation (insertion, deletion and substitution) used to transform one string of text into another ( 27 ). Visualising the resulting distance in a matrix allows us to rapidly visually compare all participants against each other and detect any outliers, or participants that appear to use similar visualisation strategies. The darker the shade of red the more similar the scanpaths are; the darker the shade of blue the less similar they are. Figure 2 shows an example of the Levenshtein distance for the 2 groups (description and no-description) for the “Rhyl Sands” painting, where the participants' scanpaths can be compared with one another in that condition and between conditions. This allows for rapid initial analysis of the spatial and sequential aspects of the participants’ eye-movements as they viewed the paintings. In this representative example we can see that most of the cells are red in both groups, implying that the sequence of transitions is fairly similar for most of the participants.

**Figure 2a.**
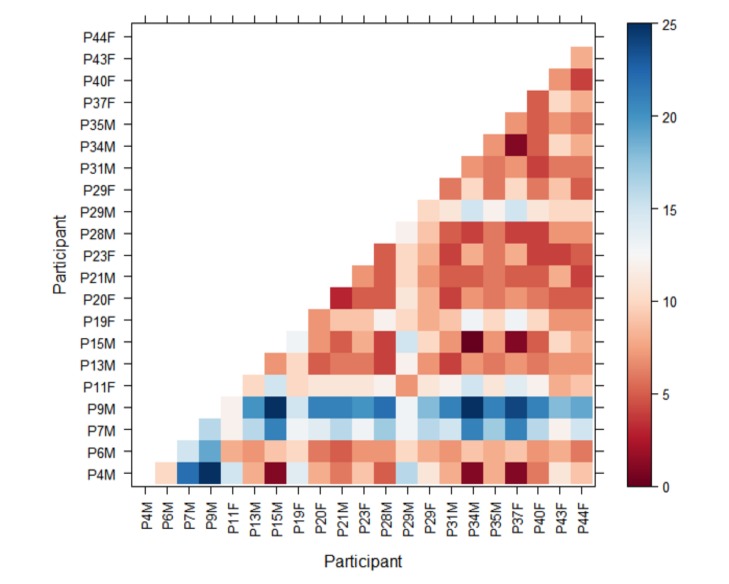
No-narrative

**Figure 2b.**
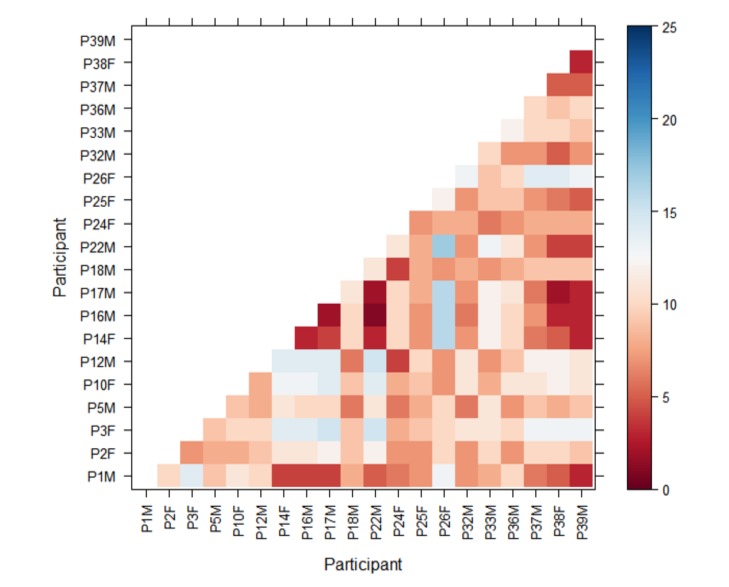
Narrative

The visualisations were generated for each stimulus for the two groups (description and no-description). The visualisations allow for rapid high level comparison of the two conditions per painting. As mentioned previously this also makes it easier to identify outliers among the participants for further examination, or possible exclusion from subsequent data analysis. A summary of the Levenshtein distance results for each painting can be seen in Table 2.

**Table 2.** Summary of Levenshtein distance per painting

	**Levenshtein distance**
**Condition 1**	*Max*	*Mean*	*SD*
Rhyl Sands	25	9.19	5.41
Flask-walk, Hampstead	20	7.88	4.09
Self-portrait	39	15.95	7.74
When the West with evening glows	28	12.36	6.82
14.6.1964	32	11.57	7.03
Woman and suspended man	40	13.15	8.28
Sir Gregory Page-Turner	32	14.77	7.60
Release	30	14.67	7.90
**Condition 2**			
Rhyl Sands	18	8.57	3.84
Flask-walk, Hampstead	35	18.23	6.45
Self-portrait	33	17.23	9.81
When the West with evening glows	28	14.24	5.74
14.6.1964	33	12.25	6.44
Woman and suspended man	31	15.12	7.72
Sir Gregory Page-Turner	33	16.78	7.29
Release	30	13.94	6.47

## Results

**Figure 5.**
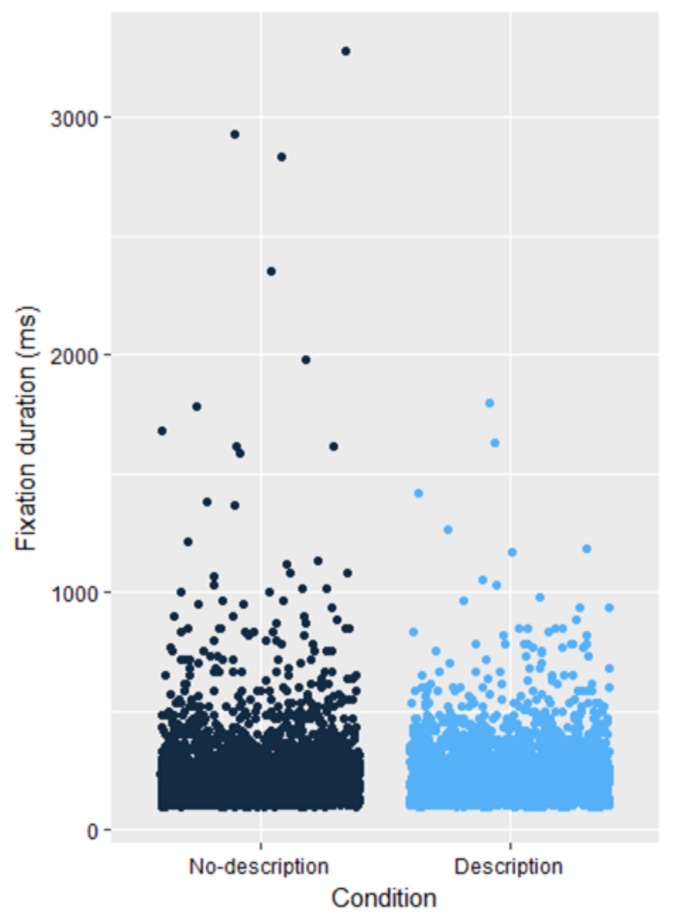
Fixations for both groups with their associated durations (all paintings)

The results indicate that the majority of fixations made for both groups tend to occur in the 100-300 ms duration range, suggesting that relatively short fixations are predominant in both conditions. There were, on average, 893 (SD = 72) fixations in the no-description group and 815 (SD = 97) fixations in the description group. The mean fixation duration for the no-description group was 239 ms (SD = 191) and 227 ms for the description group (SD = 128). Figure 5 shows the fixations for both conditions along with the fixation durations. A two-way mixed ANOVA with trimmed means (ɣ = 0.2) which was used due to data violating parametric assumptions ( 28, 29 ), showed that there was no significant difference between fixation counts for the description and nodescription groups. There was a significant but small main effect of painting (Q = 4.15, p = .006, ɳ² = .04), which post-hoc pairwise t-tests (Bonferroni correction) showed was significant for “Flask-walk, Hampstead” and “Sir Gregory Page-Turner” paintings (p = .013).

**Figure 6.**
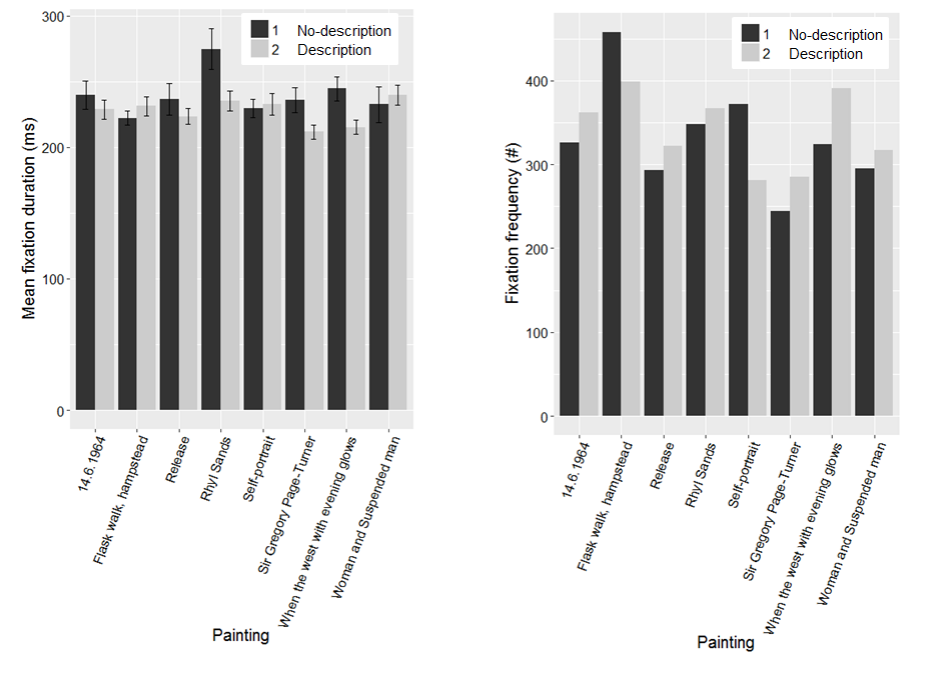
Distribution of distance results of permutation test for the painting “Release”. The vertical bar shows the distance for the description and no-description conditions

Figure 6 summarises the average fixation durations and number of fixations for each paining for both groups.

**Figure 7.**
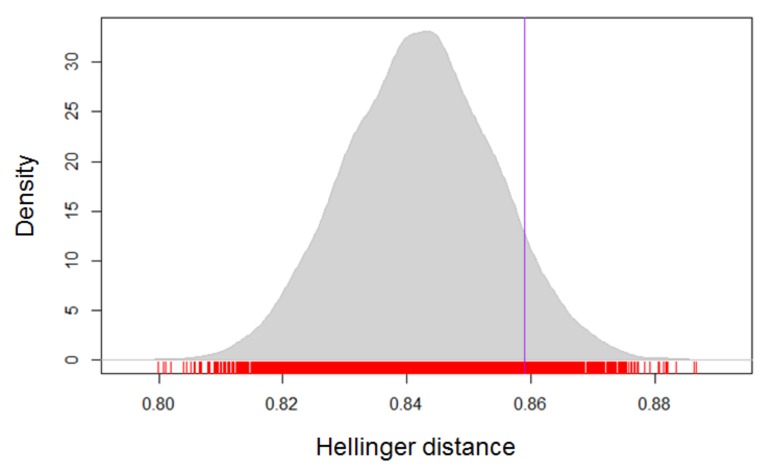
Distribution of distance results of permutation test for the painting “Release”. The vertical bar shows the distance for the description and no-description conditions

The results of the permutation test, with the exception of the painting “Release” did not show any significant differences in transitions between the groups, resulting in p values greater than .10. The painting “Release” did indicate a difference between groups (Hd = 0.859, p = .08), see Figure 7. Although this is not significant for α = .05.

This form of analysis is known to be very robust, and there is thus a very low risk of a type I error.

We examined the recording quality between the two groups to see if this could have impacted on any differences between the groups. Recording quality pertains to a percentage value that is derived from the number of gaze samples identified by the eye-tracking software that is divided by the number of attempts. Problems arising from a failure to detect the eyes and participants looking away from the screen can all contribute to reducing the recording quality value. To this end a t-test was carried out comparing the recording quality percentage values for both groups. The difference was not significant t(37) = 0.61, p > .05 suggesting that any differences detected were due to behavioural factors rather than as a result of uneven recording quality between the groups. 

## Discussion

The majority of the paintings (n=7) did not demonstrate any difference in terms of fixation and transition behaviour. The painting that was associated with a difference between groups was “Release”. As the permutation test is robust against type I error ( 26 ), this result suggests that there was a real difference between the groups for this painting.

It is notable that this painting lacks distinctive features differentiating one area of the painting from any other. The text, which provided an explanation of what the features of the painting represent (images of chromosomes viewed through an electron microscope) could explain why transitional behaviour differs between the groups in this case. Here, the description appeared to provide information that could not be gleaned from the painting itself, and it thus caused people to examine the features of the painting differently. With no salient features and a fairly uniform pattern, there may not otherwise be cues to drive viewing behaviour.

68% of the participants who read the description thought that it did make a difference to how they subsequently viewed the painting. A difference in gaze behaviour was not observed between groups in this experiment, and it would thus be interesting to further explore the potential nature of this difference, if it exists.

Recently there has been a move towards providing descriptive information in a form that departs from formal language, using instead descriptions that focus on placing the work in context and describing the artist's intentions ( 30 ). The work reported here focuses on detecting whether reading a descriptive text leads to a difference in visual behaviour; future work could systematically address how different forms and formats of curatorial narrative affect gaze patterns as well as different types of image.

The method presented here goes some way toward removing biases that can be introduced by researchers when manually defining areas/regions of interest. Although in the current study it was applied to detecting differences in visual behaviour with paintings, the method could also be applied to other domains. Applying a grid to the stimulus allows for comparison between these uniform spatial Units of Interests (UOIs), defined using a data-driven approach. This could be applied to any type of stimulus that lacks obvious or predefined semantic areas or regions. AOIs are typically added to segment a stimulus in response to a hypothesis. Changing the AOI changes the hypothesis, and adding AOIs after recording data thus equates to formulating a post-hoc hypothesis ( 6 ). Data-driven UOIs allow the segmentation of the stimuli space into equally sized regions based on the clustering of the fixation data. This allows for the easy comparison of such UOIs to determine areas of the stimulus that the participants focus most attention on, and how they move between these areas. This data-driven method allows an unbiased, exploratory approach to interpreting gaze data across a stimulus. To mitigate the arbitrary selection of grid size when using a gridded AOI system, Holmqvist et al. ( 6 ) recommends several different cell sizes be used. As changing the AOI size has an effect on the results, there may be a temptation for researches to do this until they find a statistically significant result, a practice that can be problematic from a scientific perspective ( 18 ). The method presented in this work addresses this issue by using a combination of clusters within the gaze data and pragmatic considerations to determine the size of the grid.

### Limitations

A primary limitation of this study from the perspective of the domain was the fact that participants were not able to view the descriptive text and painting simultaneously or switch between them, as they would on a website or in a gallery; separating them was necessary to ensure the paintings could be presented in the same way to participants in both conditions. Paintings were viewed for only 10 seconds, and it may thus be the case that, regardless of the description, in this short time the eyes were instinctively drawn to the salient components of the painting, such as buildings, body shapes, facial details etc (bottom-up features). The texts used here were functional, and used for describing an artwork to aid its identification; whilst we can hypothesize other forms of curatorial narrative will not affect viewing behaviour, we cannot be sure. Viewing a painting online is likely to be quite different to viewing in a gallery, where scale and context will affect the experience, so it is not clear whether these results would extend to this scenario.

### Conclusions and future work

The use of a grid based system with cell size determined by data-driven clustering allows for the creation of units of interest (UOIs), which can serve as a basis for subsequent analysis. The UOIs simultaneously aid in removing the bias introduced by researchers deciding on the size and location of these areas, and allow direct comparison between the units, as they are of equal dimensions. The study demonstrated that viewing a descriptive text has no significant impact on subsequent gaze patterns over a painting, with one exception -- an image that had a relatively uniform pattern with few distinctive features. The effect may also vary according to the type and quality of the textual information provided in these descriptions, and it would be interesting to test this in a future study. Here we examined descriptions -- it may be that different forms of curatorial narrative have a greater affect on viewing patterns. The techniques described in this study may have a much wider application, as they could also be of use in identifying the effects of descriptive data on viewing behaviour in other domains, such as understanding how patient history shown before a subsequent medical scan affects the way this image is viewed.

### Ethics and Conflict of Interest

The authors declare that the contents of the article are in agreement with the ethics described in http://biblio.unibe.ch/portale/elibrary/BOP/jemr/ethics.html and that there is no conflict of interest regarding the publication of this paper.

### Acknowledgements

We would like to thank the Engineering and Physical Sciences Research Council (EPSRC) for funding this work through grants EP/K502947/1, EP/L504877/1 and EP/K503782/1-079. We would also like to thank Liz Mitchell and Alex Wood for their help selecting the paintings.
